# Randomised Comparison of Culotte- versus Double Kissing Crush Stenting in de novo non-left Main Coronary Bifurcation Lesions: Rationale and Design of the Bifurcation Bad Krozingen trial-3 (BBK-3)

**DOI:** 10.1007/s12265-025-10626-x

**Published:** 2025-05-27

**Authors:** Faridun Rahimi, Nikolaus Löffelhardt, Jan Minners, Philipp Breitbart, Kilian Franke, Tau Sarra Hartikainen, Christian Valina, Constantin v. Zur Mühlen, Thomas Nührenberg, Adnan Kastrati, Felix Woitek, Albrecht Elsaesser, Mohamed Abdel-Wahab, Samuel Sossalla, Willibald Hochholzer, Dirk Westermann, Franz-Josef Neumann, Christoph Olivier, Miroslaw Ferenc

**Affiliations:** 1 Department of Cardiology and Angiology, Medical Center, Faculty of Medicine, University of Freiburg, University of Freiburg, Suedring 15, 79189 Bad Krozingen, Germany; 2 Department of Cardiology, Technische Universitaet Muenchen, Deutsches Herzzentrum Muenchen, Munich, Germany; 3 Department of Internal Medicine and Cardiology, Heart Center Dresden, University Hospital, Technische Universität Dresden, Dresden, Germany; 4 Heart-Circulation Centre, Clinic Oldenburg, Oldenburg, Germany; 5 Heart Center Leipzig, University of Leipzig, Leipzig, Germany; 6 Department of Cardiology, University Hospital Giessen Kerckhoff Clinic, Giessen, Germany; 7 Department of Cardiology and Intensive Care Medicine, Klinikum Wuerzburg Mitte, Würzburg, Germany

**Keywords:** Bifurcation, 2-stent strategy, Culotte, DK-crush, PCI

## Abstract

Whether culotte or double kissing (DK)-crush stenting is the superior systematic 2-stent technique in non-left main bifurcation lesions is unclear. The BBK-3 (unique identifier NCT 04192760) is a multicenter, prospective, randomized controlled trial to test the hypothesis that culotte stenting reduces maximal percent diameter restenosis at 9-month follow-up compared to DK-crush stenting in de-novo non-left main coronary bifurcation lesions using approved, third-generation drug-eluting stents (DES). A total of 400 patients will be randomized in a 1:1 ratio to receive a two-stent strategy. The primary study endpoint is the maximal percent diameter in-stent restenosis within the bifurcation at 9 months, assessed by quantitative coronary angiography. Secondary endpoints include target lesion revascularization (TLR), the composite of death and myocardial infarction, emergent cardiac bypass surgery and TLR (MACE) at 12 months. BBK-3 will assist in the identification of the preferable, contemporary 2-stent strategy in the treatment of de-novo non-left main coronary bifurcation lesions.

**Graphical Abstract:**

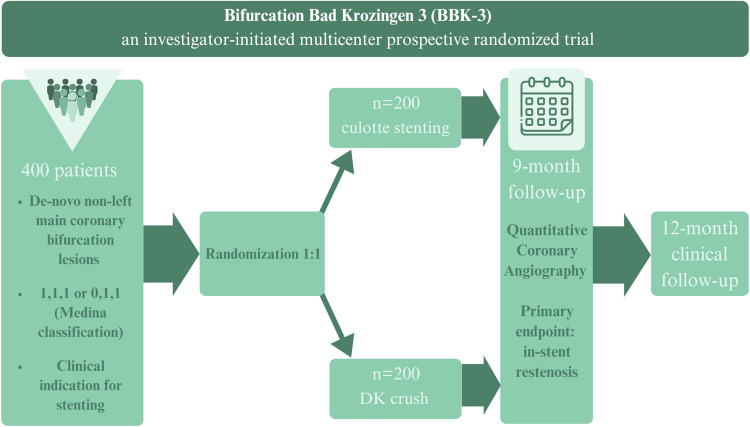

## Introduction

The optimal treatment strategy for coronary bifurcation lesions remains a subject of debate. Current guidelines and position papers recommend provisional side-branch stenting as the standard procedure for PCI in bifurcations lesions [1, 2]. However, this approach has been questioned based on improvements in stent design and focus on complex anatomies including true bifurcations lesions (Medina classification 1,1,1 and 0,1,1). Consequently, an advantage of a systematic 2-stent strategy has been demonstrated for selected left main and non-left main bifurcation lesions [3, 4]. There is still a gap in evidence regarding the most appropriate stenting technique. For instance, in the BBK-2 study, culotte-stenting demonstrated benefit over TAP [5] and the DK (double kissing)-CRUSH III study showed superiority for DK-crush as compared to culotte for distal left main bifurcation stenosis [6, 7]. However, in vitro, culotte (both with and without a DK manoeuvre) improved stent apposition in the bifurcation segment compared to DK-crush [8]. Finally, in a recent retrospective analysis investigating true non-left main bifurcation lesions, DK-culotte was associated with lower target lesion failure (TLF) rates compared to DK-crush [9]. We therefore designed the randomized BBK-3 study, which will compare culotte-stenting with DK-crush stenting in true non-left main bifurcation lesions using the newest generation of drug eluting stents (DES).

## Methods and Results

### Study Organization

The BBK-3 study (ClinicalTrial.gov unique identifier NCT 04192760) is an investigator-initiated trial, designed and conducted by the University Heart Center Freiburg · Bad Krozingen. The academic research institution Cardiovascular Center for Clinical Research (CCRC) at the University Heart Center Freiburg · Bad Krozingen supported trial coordination and management. Boston Scientific (BSI, Marlborough, US) provided partial financial support. The study protocol adheres to the Declaration of Helsinki and was approved by the institutional ethic committees at each participating center. Each subject will provide written informed consent prior to entering the study.

### Study Hypothesis

The study is designed to investigate whether culotte stenting compared with DK-crush stenting in non-left main coronary bifurcation lesions reduces maximal percent diameter stenosis at the bifurcation at 9-month follow-up.

### Study Design, Population, and Randomization

**Fig. 1:**
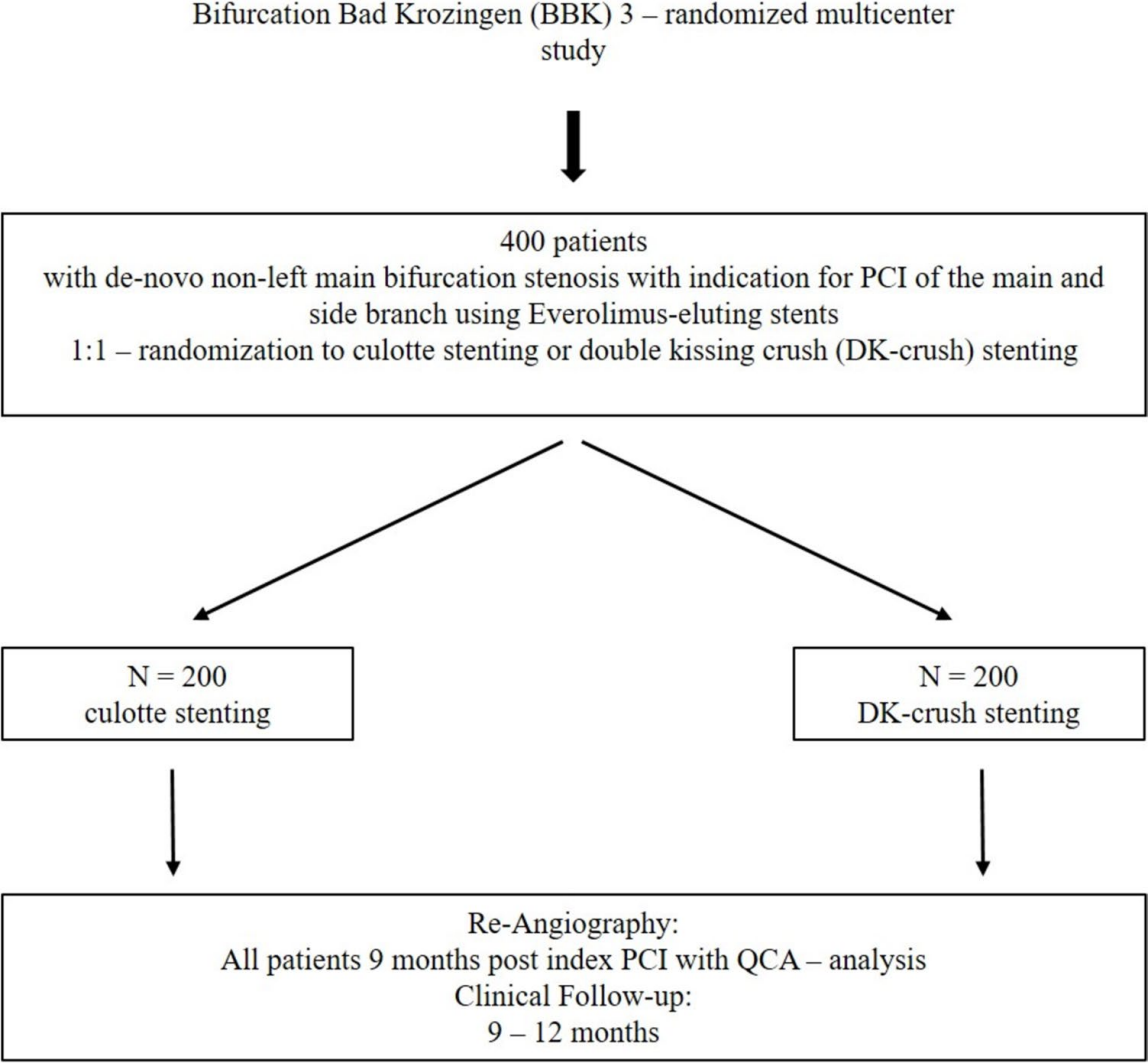
Study flow chart of the BBK 3 study; QCA, quantitative coronary angiography

The BBK-3 trial is a prospective, randomized, multicenter trial and will be conducted at 12 hospitals in Germany. Patients with a significant true de-novo coronary bifurcation lesion (Medina 1,1,1 or 0,1,1) and the clinical need for treatment will be included in the study. A total of 400 patients will be randomized in a 1:1 ratio to receive either culotte stenting or DK-crush stenting using computer-generated random sequences stratified by investigation sites. The study flowchart is shown in Fig. 1. Random permuted blocks will be employed to ensure approximate balance of treatment allocation within each site.

### Inclusion and Exclusion Criteria

Patients ≥ 18 years of age with a clinical indication, evidenced by angina/angina-equivalent symptoms or documented ischemia (non-invasive imaging such as scintigraphy, stress cardiac magnetic resonance imaging (stress-MRI) or stress-echocardiography; fractional flow reserve (FFR) or instantaneous wave-free ratio (iwFR)) or patients with acute coronary syndrome (unstable angina and non-ST-elevation myocardial infarction) are evaluated for enrolment in this trial. A de-novo non-left main true coronary bifurcation lesion – 1,1,1 or 0,1,1 according to the Medina classification – of a native coronary artery must be present with the following reference vessel diameter: main branch > 2,5 mm; side branch > 2,25 mm. The difference between vessel diameter of the main and side branch must not be larger than ≤ 1 mm. The target lesion has not been previously treated and the target vessel must be amenable to stent implantation. Patients with an acute ST-elevation myocardial infarction or patients with a life-expectancy of less than 1 year will be excluded. A complete overview of inclusion and exclusion criteria is shown in Table 1.

**Table 1:** Inclusion and exclusion criteria

**Inclusion Criteria** • Clinical indication, evidenced by angina/angina-equivalent symptoms or documented ischemia (non-invasive imaging such as scintigraphy, stress- MRI or stressechocardiography; FFR or iwFR) or patients with non ST-segment elevation acute coronary syndrome (NSTE-ACS) • Clinical indication to perform a 2-stent strategy only with Synergy™ stents for a clinically significant bifurcation stenosis as judged by the operator • De-novo non-left main coronary bifurcation lesions - 1,1,1 or 0,1,1 according to the Medina classification - of a native coronary artery with the following reference vessel diameters: main branch > 2,5 mm; side branch > 2,25 mm. The difference between vessel diameter of the main and side branch is ≤ 1 mm • The target lesion has not been previously treated with any interventional procedure • The target vessel (main branch and side branch) must appear feasible for stent implantation • Patient has no other coronary intervention planned within 30 days of the procedure • Patient has been informed of the nature of the study and agrees to its provisions and has written informed consent as approved by the Ethics Committee Patient is willing to comply with all required post-procedure follow-up
**Exclusion Criteria** • Patient had an acute ST-elevation myocardial infarction within 72 hours preceding the index procedure or target vessel contains intraluminal thrombus • Use of any other coronary stent than Synergy™ and Synergy Megatron™ except for bail-out situations • Patient with a known hypersensitivity or contraindication to the needed antithrombotic therapy, stent type or contrast media that cannot be adequately pre-medicated • Non successful treatment of other lesion during the same procedure • Patient with a severe bleeding diathesis, history of recent major bleeding or stroke (≤ 6 months), coagulopathy or severe liver disease • Patient has a co-morbidity (i.e. cancer) that may cause the patient to be non-compliant with the protocol, or is associated with limited life-expectancy (less than 1 year) • Patient is participating in any other clinical study with an investigational product • Patient is known to be pregnant or lactating at time of inclusion

*MRI* magnetic resonance imaging, *FFR* fractional flow reserve, *iwFR* instanstaneous wave-free ratio, *NSTE-ACS* non ST-segment elevation acute coronary syndrome

### Percutaneous Coronary Intervention

Site investigators are board-certified cardiologists. Each investigator involved in the study will be trained on the study protocol and the intervention techniques to ensure uniformity of study procedures. Arterial access is obtained either via the radial or the femoral artery. The size of the sheath for the procedure is left to the discretion of the interventionist. An activated clotting time (ACT) of more than 250 s is recommended during the procedure. Approved Everolimus-eluting stents will be used for bifurcation stenting (Synergy™, Boston Scientific). The culotte stenting and DK-crush stenting procedures have been described in detail previously [10]. A brief explanation is given below. For both techniques, lesion preparation in the main vessel and side branch may be undertaken according to operator preference.

#### Culotte-Technique

Culotte stenting has to be performed according to the European Bifurcation Club white paper on stenting techniques for patients with bifurcated coronary artery lesions [10]. In brief, after lesion preparation, the side branch has to be stented first. The first stent is placed from the main branch into the side branch, covering the entire diseased segment with a wire jailed in the main vessel. After removal of the jailed wire the main vessel is rewired through the stent struts and is dilated with a balloon to separate stent struts. The side branch wire is then removed and the main vessel is stented covering the proximal and distal segment. After a proximal optimization technique (POT)-manoeuvre the side branch is re-wired and a kissing-balloon post-dilatation with non-compliant balloons is performed. Balloon sizing should be in accordance with the diameter of the vessels itself (1:1 ratio). Finally, a POT-manoeuvre is performed.

#### DK-Crush-Technique

DK-Crush stenting has to be performed according to the European Bifurcation Club white paper on stenting techniques for patients with bifurcated coronary artery lesions [10]. In brief, after lesion preparation the side branch is stented first. Side branch stent should have a small protrusion into the main branch. Before stent implantation in the side branch, an adequately sized balloon should be placed in the main branch, just opposite to the side branch ostium. After side branch stent implantation stent-balloon and wire are removed and the balloon of the main branch must be inflated to crush the proximal stent struts against the ostium and the vessel wall. The next step is to rewire the side branch and to perform a first kissing balloon manoeuvre. After removal of the side branch wire, a stent is implanted into the main branch followed by a second re-wiring of the side branch and a second kissing balloon procedure. The procedure ends with a final POT.

### Coronary Imaging

#### Intracoronary Imaging

Intracoronary imaging with intravascular ultrasound (IVUS) or optical coherence tomography (OCT) is not mandatory. However, its use is recommended for complex interventions.

#### Coronary Angiography

Before starting the intervention, the bifurcation stenosis is visualised angiographically in two different projections. The bifurcation must be clearly visible without pronounced perspective foreshortening. The angulation of the two projections must be far apart, provided that good quality visualisation of the bifurcation is possible. After the procedure, the bifurcation is visualised angiographically in the same two predefined projections. Identical projections will be used in the control coronary angiography performed 9 months later to assess the bifurcation.

### Follow-up

All patients enrolled in the study will be required to complete the follow-up to evaluate long-term results.

#### Angiographic Follow-up

All patients will be scheduled for repeat angiography at 9 months (± 30 days) after the index procedure. Angiographies will be performed as described in the predefined projections to visualise the treated bifurcation. All angiographies, including unscheduled angiograms, will be analysed by an independent angiographic core laboratory (coreLab Black Forest GmbH, Bad Krozingen, Germany). For quantitative coronary angiography, angiograms obtained at baseline, at completion of the intervention, and at 9 months follow-up (in case of an unplanned coronary angiography before 9 month) will be analysed using a computer based system dedicated to bifurcation analysis (Qangio XA, version7.0, Medis, Leide, Netherlands), according to the standard operating procedure of the angiographic core laboratory. Quantitative angiographic measurements will be obtained of the three segments of the bifurcation lesion: the proximal and distal segment of the main branch and the side branch. Measurements in the stented portion of the vessel (in-stent) and in the distal or proximal 5 mm margin (edge) will be performed.

#### Clinical Follow-up

Patients will undergo clinical follow-up 9–12 months post procedure (mainly in person; if not possible by telephone contact or contact with general practitioner). An option to extend clinical follow-up to 3 years is being pursued.

### Study Endpoints

The primary endpoint is the maximal percent diameter in-stent restenosis within the bifurcation at 9 months, assessed by quantitative coronary angiography. Key secondary endpoints are binary restenosis (≥ 50% diameter stenosis) rate at any segment of the bifurcation at 9 months post procedure (estimated by Quantitative Coronary Angiography (QCA) analysis), Target Lesion Revascularization (TLR), Freedom from Major Adverse Cardiac Events (MACE) and the rate of stent thrombosis according to the definition of the Academic Research Consortium (ARC definition). A complete list of all endpoints is provided in Table 2.

**Table 2:** Study endpoints

**Primary study endpoint** • Maximal percent diameter stenosis at the bifurcation at 9 months
**Secondary endpoints** • Binary restenosis (≥ 50% diameter stenosis) rate at any segment of the bifurcation at 9 months post procedure • Binary restenosis (≥ 50% diameter stenosis) in the main and side branch at 9 months post procedure • TLR of the main and side branch at 9–12 months post procedure. Intervening target-lesion revascularization is defined as any repeated percutaneous revascularization of the stented segment, including the 5-mm proximal and distal margins • MACE defined as death, myocardial infarction (according to the fourth universal definition of myocardial infarction, 2018), emergent cardiac bypass surgery, or TLR at 9–12 months • Device success defined as attainment of < 30% residual stenosis of the target lesion using drug-eluting stent (DES) in the main and side branch • Procedure time, radiation time and volume of used contrast medium • Post-procedure thrombotic stent occlusion at 9–12 months • Disabling stroke defined as stroke requiring inpatient rehabilitation or skilled nursing care • Bleeding classified as type 3–5 according to the BARC classification

*TLR* target lesion revascularization, *MACE* major adverse cardiac event, *BARC* the bleeding academic research consortium

### Sample Size Calculation and Statistics

The study is designed to have an 80% power to detect a 25% relative reduction of the primary endpoint by culotte technique as compared with DK-crush stenting at a significance level of 0.05. Based on our previous study we assume a maximum percent diameter stenosis of 20% in the culotte arm and a common standard deviation of 22% [5]. A sample size of 172 patients in each arm is required to detect a reduction in maximum percent diameter stenosis from 26.7% to 20% by culotte stenting as compared with DK-crush stenting with 80% power. The study will include 200 patients in each arm to allow for losses to angiographic follow-up. A modified intention-to-treat analysis of angiographic outcome measures will be performed, including the primary endpoint, which is restricted to patients with follow-up angiography. Clinical endpoints will be analyzed according to the intention-to-treat principle. Discrete variables will be reported as counts (percentages) and continuous variables as mean ± standard deviation. For discrete variables, we will test differences between groups with the chi-square test or Fisher exact test when expected cell sizes are less than five. Two-tailed t-tests will be used to compare continuous variables. As a sensitivity analysis, we will also perform analysis of covariance with pertinent baseline variables as covariates, to corroborate our primary analysis. All tests are two-sided and statistically significance is considered at 5%.

### Organization/Ethical Concerns

This clinical trial was designed and recorded with accordance to the ICH-GCP, with applicable local regulations (including European Directive 2001/20 EC), and with the ethical principles laid down in the Declaration of Helsinki. The protocol and the proposed informed consent form have been reviewed and approved by a properly constituted Independent Ethical Committee (IEC) before trial start. A signed and dated statement that the protocol and informed consent have been approved by the IEC must be available prior to initiation of the trial.

### Current Study Status

The BBK 3 trial started recruiting in August 2020. As of February 2025, 356 patients were included in the study. The anticipated completion of inclusion is May 2025 and the anticipated completion of the study is May 2026.

### Sub-group Analyses

The following pre-specified sub-group analyses are planned. The primary endpoint will be further analysed according to age, sex, diabetes, kidney function, calcification grade, cardiac biomarkers, and DEFINITION score [11].

## Discussion

The primary hypothesis of the BBK-3 trial is that culotte stenting is superior to DK-crush stenting and reduces maximal percent diameter restenosis at 9-month follow-up in patients with true, non-main stem bifurcation lesions.

Stenting of coronary bifurcation lesions is associated with suboptimal clinical results including more frequent restenosis and stent thrombosis compared with non-bifurcation lesions [12]. Therefore the European guidelines and the European Bifurcation Club (EBC) consensus advise a stepwise provisional approach with optional two-stent strategy [1, 2]. This recommendation is based on a patient-level pooled analysis of two large trials and two study-level meta-analysis, showing improved survival for a provisional single-stent approach as compared with systematic dual stenting [12–14]. This paradigm has been challenged by studies analysing the treatment of true bifurcation lesions. The DKCRUSH-II study in true bifurcation lesions was the first to demonstrate superiority of the two-stent approach as compared with provisional side-branch stenting during 5-year follow-up regarding to target lesion revascularization (TLR) [3, 15]. Subsequently, in the DKCRUSH-V study, which focused on true left main bifurcation lesions, the two-stent strategy reduced the risk of TLF at 1 year and also during 3-year follow-up compared to provisional stenting [4, 16].

At least three developments may account for the discrepant results of the older compared to the more recent data, namely stent design, lesion selection, and stent technique. In the early studies, which established the concept of provisional side branch stenting, mostly first-generation DES were used, whereas in the more recent studies, supporting dual stenting, new-generation thin-strut DES were employed. In the Nordic-Baltic Bifurcation IV study in particular, the trend towards an advantage of the two-stent approach was more pronounced with new-generation DES than with first-generation DES [17].

Furthermore, the selection of the lesion is of decisive importance. Even with the standard strategy of provisional stenting, a considerable number of side branches are stented (5–20% in randomized trials). Especially in true bifurcation lesions with long and high-grade stenoses in the side branch, the probability of a successful single-stent strategy is lower [18]. The more recent studies are suggesting a benefit of systemic dual stenting of true bifurcation lesions as categorised by the Medina classification (Medina 1,1,1 and 0,1,1). Metaanalyses that contributed to the recommendation of provisional stenting for bifurcations included studies in which also non-true bifurcation lesions were present. That might have influenced the results to the detriment of the two-stent strategy [12, 13].

It is still unclear, which stenting technique should be favoured in bifurcation lesions. Most of the evidence for systematic 2-stent strategy in true or complex bifurcations comes from DK-crush stenting and the majority of DK-crush studies were carried out in centers with a long tradition of this intervention technique. There may be differences in outcome depending on the technique of double stent implantation. In BBK-2 culotte stenting was superior to TAP stenting in a cohort with mostly non-left main bifurcations [5]. The DKCRUSH-III trial demonstrated an advantage for the DK-crush technique compared to culotte stent implantation for left main bifurcations in terms of lower TLF [7]. It remains unclear, whether these findings pertain to bifurcations other than the distal left main stem owing to smaller vessel size and lower bifurcation angles [19]. In addition, comparison of the instent restenosis rates of the DK-crush group from the DK-CRUSH II trial with the culotte group from BBK 2 shows a 25% lower rate in favour of culotte stenting. The culotte stenting procedure was recently modified by an additional kissing manoeuvre with the aim of achieving better stent coverage of the bifurcation. This DK-culotte technique was tested in a bench test against the “classical” culotte and DK-crush. It showed superior results for DK-culotte and culotte in terms of stent apposition in the bifurcation segment compared with DK-crush [8]. A recently published retrospective analysis showed that DK-culotte was associated with lower TLF rates compared with DK-crush in true non-left main bifurcation lesions [9]. However, a direct, prospective comparison of the culotte stenting technique with DK-crush in non-left main stenoses in a prospective analysis is not yet available.

Thus, we designed the BBK-3 study to be conducted in centers with experience in both stenting techniques. Only patients with non-left main true bifurcation lesions are included, who present with either typical angina pectoris, an acute coronary syndrome (except ST elevation myocardial infarction), or invasive or non-invasive evidence of ischaemia. The same criteria apply to re-interventions after the index procedure corresponding to the current guidelines for myocardial revascularisation. The newest generation of drug-eluting stent is used for this purpose. All necessary devices can be used for lesion preparation, as well as intravascular imaging to optimize the procedure and verify the result. Since at initiation of the study the level of recommendation for intravascular imaging was low, its use was left to the decision of the interventionalist. Culotte and DK-crush stenting are compared in a randomised fashion and instent restenosis (primary endpoint) is examined by control coronary angiography using predefined standardised imaging projections. Based on the results of this trial with instent restenosis as primary endpoint a subsequent trial investigating a clinical endpoint (requiring a substantially higher number of patients) is planned.

## Conclusions

The BBK-3 study will compare culotte with DK-crush stenting in non-left main bifurcation stenosis based on the hypothesis that using the newest-generation DES culotte stenting reduces maximal percent diameter restenosis at 9-month follow-up compared to DK-crush stenting.

### Limitation

Since only the Synergy™ stent was used the results of the current trial cannot be generalized across all 3rd generation DES.

### Clinical Relevance

BBK-3 will provide important information on whether DK-crush or culotte is the preferred 2-stent strategy for true non-left main bifurcation lesions.

## Data Availability

The datasets analysed during the current study are available from the corresponding author on reasonable request.

## Abbreviations

BARC: The bleeding academic research consortium
BBK trial: Bifurcation Bad Krozingen trial
DES: Drug-eluting stent
DK: Double kissing
FFR: Fractional flow reserve
IEC: Independent Ethical Committee
ICH-GCP: International Council for Harmonisation-good clinical practice
iwFR: Instantaneous wave-free ratio
MACE: Major adverse cardiac event
MRI: Magnetic resonance imaging
NSTE-ACS: Non-ST-segment elevation acute coronary syndrome
PCI: Percutaneous coronary intervention
POT: Proximal optimization technique
TAP: T and protrusion
TLF: Target lesion failure
TLR: Target lesion revascularization
